# Methods for Rapid Characterization of Tunable Microbubble Formulations

**DOI:** 10.3390/bioengineering11121224

**Published:** 2024-12-03

**Authors:** Savannah L. Harpster, Alexandra M. Piñeiro, Joyce Y. Wong

**Affiliations:** 1 Department of Biomedical Engineering, Boston University, Boston, MA 02215, USA; harpster@bu.edu (S.L.H.);; 2 Division of Materials Science & Engineering, Boston University, Boston, MA 02215, USA

**Keywords:** microbubbles, ultrasound, particle segmentation, tissue-mimicking phantom, medical imaging, lipids

## Abstract

To optimize microbubble formulations for clinical applications, the size distribution, concentration, and acoustic intensity must be rapidly measurable to allow for the successful iteration of microbubble design. In this paper, a comprehensive method was developed to compare microbubble formulations with different lipid shell compositions using optical and acoustic methods of measurement to collect the size distribution, concentration, and mean scattering intensity. An open-source ImageJ macro code was modified for the selective counting and sizing of brightfield microbubble images. A high-throughput agarose phantom was designed to collect multiple scattering reflections of microbubble samples to estimate the echogenicity of each microbubble solution. The information contained in the size distribution and concentration, combined with the instantaneous scattering power, can identify modifications needed for prototyping specific microbubble formulations.

## 1. Introduction

Recent innovations in the noninvasive medical imaging space include the development of microbubbles, sophisticated ultrasound imaging contrast agents with gas-filled cores. Microbubbles are particles with diameters in the micron (1 µm) range. Their size and gas core provide enhanced contrast, as the resonant frequency of most microbubbles falls within the range of standard medical ultrasound machines, around 1–10 MHz [1]. Microbubbles compress and produce ultrasound backscatter reflections due to radial shell oscillations significantly greater in magnitude than the surrounding incompressible tissue environment. This detectable difference in attenuation proved useful to clinicians, and the rapid development of several microbubble formulations were FDA-approved in the 1990s [1]. Commercially available microbubbles for clinical ultrasound imaging are nontargeted, dissipate quickly after administration, and are often used for measuring perfusion.

Microbubbles can be produced using a variety of methods, with varying components, compositions, and approaches to manufacturing. The production method of microbubbles has an influence on their size, stability, and, ultimately, clinical outcome. Microfluidic devices are used to generate a monodisperse microbubble population while simpler, FDA-approved formulations are often polydisperse [2]. Current clinical formulations are simple in their design and often involve a monolayered ‘shell’ with a gas core. Depending on the application, components comprising the shell and the core can be modified to increase particle stability over time. Perfluorocarbon gases are often used as they provide superior stability compared to oxygen or nitrous oxide [2,3]. Microbubble shells typically comprise polymers, proteins, or lipids. These can be modified to include polymer brushes, targeting ligands, or crosslinking molecules for specific applications. Depending on the architecture of the outer shell environment, inherent properties, such as acoustic scattering power and stability, may be altered to impact the nonlinear behavior of microbubbles.

A single-bubble design has been considered in the literature for targeted microbubbles but has faced challenges in clinical translation [4,5]. In contrast, generic, FDA-approved microbubbles are biologically inert, dissolve rapidly, and produce a detectable acoustic backscatter signal. This predictable signal relies on the inherent nonlinear oscillation behavior of the shell under acoustic pressure relative to its soft tissue surroundings. However, any modifications, e.g., adjusting the dissolution rate or biological interactivity, requires re-evaluating the microbubbles’ echogenicity to ensure acoustic performance is preserved. Additionally, as clinical applications have unique microbubble design requirements, specialized diagnostic tests must be developed to evaluate clinical efficacy and safety. For instance, the design and validation process for microbubbles used in myocardial perfusion differs from that required for targeted soft tissue imaging.

Microbubbles also have broader applications in drug delivery [1,5,6]. At higher acoustic pressures, the bubble shell ruptures, and inertial cavitation occurs; at lower acoustic pressures, the microbubble undergoes stable cavitation events by experiencing sequential radial oscillations, producing a pattern of nonlinear intensity. Microbubble cavitation (at the cell surface) has also been shown to influence sonoporation events in cells, increasing drug uptake by means of convection and transfer across the pore membrane [5,7]. It also leads to cellular events aligning with pore formation, useful for potential extracellular delivery mechanisms or for enhancing therapeutic uptake [7]. Shockwaves generated at the site of shell cavitation can perforate and damage surrounding cell membranes [8]. Molecularly targeted microbubbles for imaging applications using ultrasound are also reported in the literature, but they have not yet been successfully translated for clinical use [8,9].

To our knowledge, the only soft tissue application microbubbles for which there is FDA approval is the imaging of liver lesions using Lumason [10,11]. Microbubbles are not only used in diagnosis but also in the characterization of the degree of malignancy of the lesion [12]. Although this clinical need varies from our intended use, Lumason microbubbles for liver lesions have further proven to be a cost-effective and safer diagnostic alternative to both CT and MRI imaging [12]. The utilization of clinical ultrasound as a diagnostic tool but also a potential therapeutic tool can be expanded to a variety of clinical applications.

The challenge in translating specialized microbubbles to clinical use lies in balancing their functionality with safety and viability. The rapid evaluation of nominal bubble properties, such as size distribution, concentration, and relative acoustic scattering power, is essential when designing advanced microbubble systems with therapeutic agents or targeted ligands. Additionally, it is critical to determine the optimal ‘microbubble dosage’ for clinical ultrasound imaging while ensuring the appropriate therapeutic drug dosage is administered. Previously, we reported the ‘optimal’ microbubble shell parameters given a few tunable components; size was also controlled for using a microfluidic device [13].

Here, we describe a streamlined method for the rapid assessment of ultrasound contrast agents while taking their echogenicity into account. For the iterative design of microbubbles with custom formulations, a system that quickly assesses ultrasound signal output under clinically relevant conditions is necessary to ensure signal dampening does not occur because of reformulation. Our workflow and ultrasound multi-well phantom design can be adapted for use with any clinical ultrasound machine and single- or multielement ultrasound transducers for the acquisition of raw signal data for further parametrization.

Our workflow includes an adaptation to an open-source microbubble-specific particle segmentation code that tabulates measured particle sizes based on their inherent properties such as circularity and solidity. This allows the ability to correlate microbubble size distribution to acoustic phenomena using optical images of microbubble samples. We compare microbubble formulations using two different bulk lipids and report the resulting differences in size distribution and acoustic strength.

## 2. Materials and Methods

### 2.1. Materials

**Figure 1:**
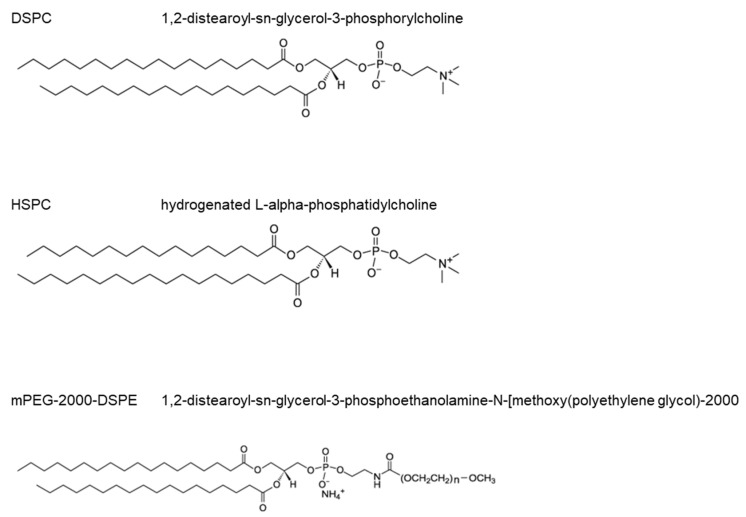
Microbubble lipid components used in microbubble formulations; 1,2- distearoyl-sn-glycerol-3-phosphorylcholine (DSPC), hydrogenated soy phosphatidylcholine (HSPC), and 1,2 -distearoyl-sn-glycerol-3-phosphoethanolamine-N[methoxy(polyethylene glycol)-2000] (mPEG2000-DSPE) are used to make microbubble formulations. Note that HSPC consists primarily of two lipid types (see Table 1). Formulations are either made with DSPC:mPEG2000-DSPE or HSPC:mPEG2000-DSPE. Mole fractions of each bulk lipid (DSPC or HSPC) used are 85% with the remaining 15% mole fraction consisting of mPEG2000-DSPE.

Distearoylphosphatidylcholine (DSPC), mPEG-2000-1,2-distearoyl-sn-glycero-3-phosphoethanolamine (mPEG2000-DSPE), and hydrogenated l-a-phosphatidylcholine (HSPC) were purchased from Avanti Polar Lipids, Alabaster, AL, USA. Octafluoropropane (OFP) gas was purchased from Advanced Specialty Gases, Reno, NV, USA. Chemical structures for each of the components used are shown in Figure 1. Ten-micron (height) CellVision chambers for single-plane imaging of microbubbles were purchased from Cell Vision Technologies, Heerhugowaard, The Netherlands. One- and three-micron-diameter polystyrene beads for size calibration were purchased from Duke Standards™ (ThermoScientific™, Fremont, CA, USA). Agarose, ethanol (200 proof) and Tris-Acetate-EDTA (TAE) buffer were purchased from Fisher Scientific (Hampton, NH, USA).

### 2.2. Microbubble Preparation and Storage

Mole fractions of the components shown in Table 1 are balanced so that each 1 mL sample contained 5.2 µmol of lipid. Briefly, lipids are weighed out and dissolved in 1 mL chloroform, and 100 µL lipid/chloroform solution are evaporated using nitrogen gas to form a lipid film at the bottom of the glass vial. The lipid film is left in a desiccator overnight and then resuspended and sonicated in a 10% glycerol, 10% 1,2-propanediol, and 80% deionized water (10:10:80) buffer solution. The solution is left to cool and then redistributed into PTFE-lined HPLC vials, where OFP gas is injected into the headspace. After the addition of OFP gas, the gas/liquid mixture is shaken with a VialMix shaker for 45 s to incorporate OFP into the microbubble core. The shaken samples are then covered with parafilm and stored at 4 °C until imaging.

### 2.3. Optical Imaging Methods and Segmentation Algorithm

Microbubbles are imaged optically with 2.5 µL of diluted microbubble solution injected into a 10 µm (height) glass slide chamber. Chambers are imaged with two different inverted microscopes: an Olympus IX83 (Olympus America, Center Valley, PA, USA), and a Zeiss Axiovert S100 (Carl Zeiss Microscopy, LLC, White Plains, NY, USA). Objectives with a 40× magnified objective are used to take brightfield images of microbubbles. Five representative images are taken from each 10 µm chamber and analyzed with ‘BubblesizerJ-2.0’, an adaptation to an existing open-source ImageJ macro developed for quantification and sizing of microbubbles [14] (Figures S1–S3). The output of ‘BubblesizerJ-2.0’ is a folder of CSV files containing tabulated data analyzed from each image. The tables of data specified for output include calculated particle area, circularity, and solidity. These parameters, specifically particle circularity and solidity, allow for further filtering so that non-microbubble objects are not counted. The ‘BubblesizerJ-2.0’ output folder can be specified in a complementary MATLAB code developed to read and plot ‘BubblesizerJ-2.0’ CSV files.

The core of the ‘BubblesizerJ-2.0’ algorithm is to filter the image such that binarization leaves only bubbles and background on the mask. From the binarized bubble image, a watershed filter is applied to further separate aggregates of bubbles into individual bubbles. The watershed algorithm is one of the key aspects to most successful particle segmentation algorithms [15].

**Figure 2:**
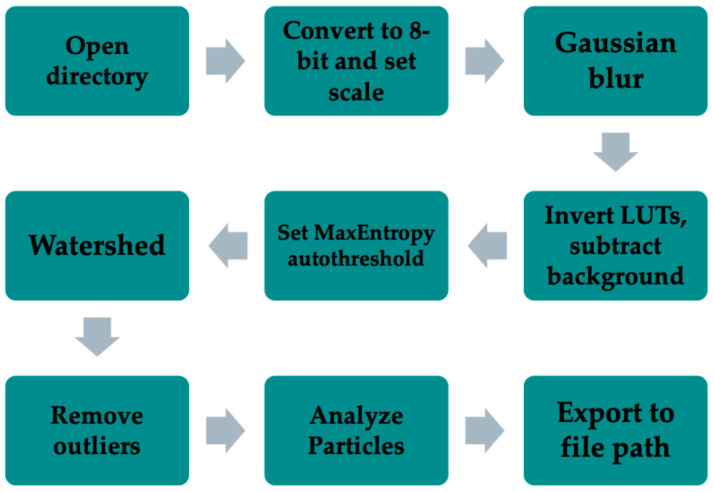
Flowchart depicts algorithm modified from original BubblesizerJ manuscript [14]. Images belonging to the specified directory are converted to 8-bit and scaled, Gaussian blurred and background subtracted, and then autothresholded using the MaxEntropy autothreshold filter in ImageJ. The resulting binarized image is then watershed filtered, extraneous outlier pixels are removed, and the ImageJ ‘Analyze Particles’ script runs and collects tabulated data to be exported to the designated output folder.

The code has been automated to run through each image in a designated file directory and export tabulated data for each image to a separate folder. An auto-threshold filter (either MaxEntropy or Otsu) minimizes bias in choosing a threshold for each image. The workflow for the modified algorithm used is shown in Figure 2.

To ensure the camera used is not distorting the final image, the measurements are calibrated using a 1 mm graticule. The algorithm is calibrated using images of varying concentrations of 1 µm- and 3 µm-diameter polystyrene beads. Images are used for determining image threshold parameters to not overly process and remove data from the image.

### 2.4. Agarose Phantom Preparation

Tissue-mimicking phantoms for ultrasound imaging are created using 1% *w*/*v* agarose gel. First, tris-acetate-EDTA (TAE) buffer is heated to around 60 °C to allow for incorporation of powdered agarose. The agarose is mixed, and the solution left magnetically stirring at a rate of 1200 rpm until cleared. To create the ‘high-throughput’ design phantom, 500 mL of TAE buffer and 5 g of agarose are mixed and set under vacuum in a Rubbermaid^®^ (Atlanta, GA, USA) container with a total volume of 763 mL. Once the solution is allowed to harden, cylinders are bored out using an 8 mm diameter core borer to create ‘wells’ for seamless side-by-side imaging of microbubble samples. When not using the phantom, it is stored in an excess of 1× TAE buffer at 4 °C with the Tupperware^®^ lid further sealed with parafilm (Orlando, FL, USA).

To reuse the phantom in multiple experiments, it can be cleaned out with ethanol and water to remove any extra lipid fragments. Excess liquid is pipetted out, and the gel block is washed twice with ethanol. Care is taken to ensure that ethanol fully penetrated each well. After washing with ethanol, two washes with 1× TAE buffer are carried out to ensure complete removal of ethanol.

### 2.5. Ultrasound Imaging and Analysis

**Figure 3:**
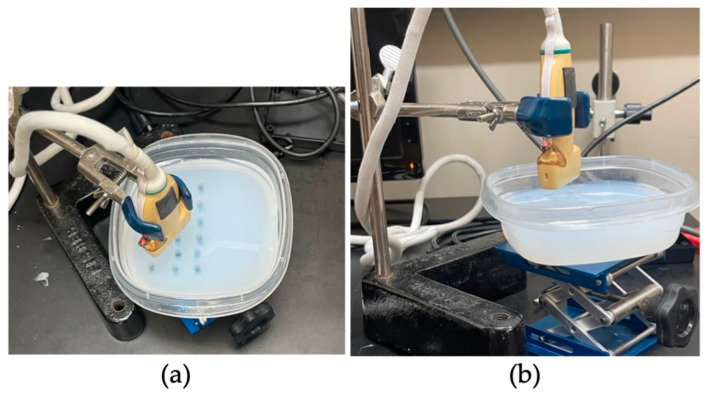
High-throughput agarose phantom for B-mode ultrasound imaging: (**a**) shows top view of agarose phantom in Tupperware^®^ container; and (**b**) shows a top-side view of agarose phantom prior to imaging.

A Terason 2000 is used with a transducer (3 to 7 MHz, 128-element linear array transducer (Model 7L3), Teratech Corporation, Burlington, MA, USA) to image our microbubbles. First, 200 mL of deionized water is degassed for 15 min prior to use in the phantom. The agarose phantom is topped off with degassed water and any excess water is removed. Next, 50 µL of microbubbles are pipetted in the center of each water-filled well, with each control well receiving a 50 µL injection of phosphate-buffered saline (PBS). Bubbles are imaged by locating the wells with the transducer positioned and stationed above the phantom (shown in Figure 3).

ImageJ is used to analyze B-mode images taken from the Terason2000 imaging system. All images are saved as 16-bit TIF files and converted to 8-bit prior to analysis. An ROI is saved from the original agarose phantom with a known diameter of 8 mm. The height of the ROI can be found from the dimensions included on the scale bar in the Terason software v. 3.6.6. To interpret the intensity output from each ‘well’, each ‘well’ is highlighted by the pre-measured ROI. Next, the mean pixel value of each well is collected, and average and standard deviation are generated across all wells of each sample type. These 8-bit greyscale intensity values are averaged across images and normalized by subtracting the mean background value from each image.

## 3. Results

### 3.1. Size Distribution and Concentration

**Figure 4:**
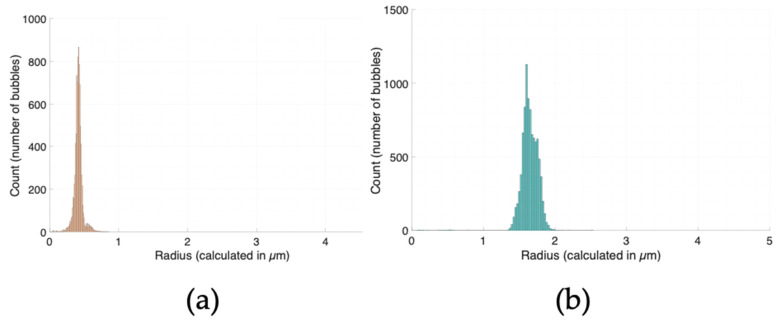
‘BubblesizerJ-2.0’ histograms for (**a**) 1 µm (0.5 µm-radius)- and (**b**) 3 µm (1.5 µm-radius)-diameter calibration beads. The estimated concentration for the 1 µm beads in Figure 4a is 3.5312 × 10^9^ ± 2.2118 × 10^8^ beads/mL (*n* = 95 bins); the estimated concentration for the 3 µm beads in Figure 4b is 3.1347 × 10^8^ ± 3.5550 × 10^7^ beads/mL (*n* = 97 bins). The average radius for Figure 4a is 0.4154 ± 0.0580 µm; the average radius for Figure 4b is 1.6472 ± 0.1197 µm. The ‘BubblesizerJ-2.0’ output was multiplied by two to obtain the particle diameter.

The population size distribution of each sample can be determined by plotting a histogram of the resulting microbubble radii values. From the volume of the sample, the microbubble concentration can be further approximated. An estimated particle concentration is calculated by a concentration equation provided by the manufacturer. The histograms and particle size population statistics for particle size standards are shown in Figure 4. Note that the vertical axis denotes the total number of objects (across all images) counted that fit in the histogram bin for that size radius. A greater number of objects counted results in a more concentrated sample when comparing the vertical axis in between the two histograms in Figure 4.

The achievable resolution depends on the volume of bubbles being imaged. The concentration approximation comes from the division of the mean particle count per image and the total volume with which those particles were imaged in. A smaller volume imaged will allow for the approximation of smaller concentrations. A 10 µm-tall chamber was used, and the volume imaged was determined from the camera pixel size and dimensions of the field of view. Although a larger volume yields a larger overall resolution, it is more consistent to use a chamber of known height. Bubbles that are deemed to be in a different plane of focus are also still picked up by the ‘BubblesizerJ-2.0’ algorithm, so under- or overcounting is not an issue.

The ‘BubblesizerJ-2.0’ algorithm calculates the number of bins used in each histogram as the square root of the total number of objects detected across all images measured. It must be noted that Figure 4a,b come from two different microscopes (Axiovert S100 and Olympus IX83, respectively), but, if the correct pixel ratio is used, no difference between the two machines was observed. Care must be taken when running the ImageJ macro and MATLAB (https://www.mathworks.com/products/matlab.html) code that the pixel size and volume dimensions are corrected to apply to the experiment.

### 3.2. High-Throughput Agarose Phantom

The advantage of using a tissue-mimicking phantom with multiple sampling wells is the rapid assessment of samples in parallel. For the development of microbubbles with a highly specialized clinical function, it is important to ensure that the bubble formulation does not dampen the inherent acoustic scattering properties of the bubbles. That is, regardless of the modular component added to the system (e.g., targeting ligand), it should not interfere with the gas and shell compression to the point of an undetectable backscatter signal. Here, ultrasound imaging was performed with a Terason imaging system, in which all B-mode images collected were processed through Terason’s beamforming algorithm. We note that intrinsic microbubble properties cannot be inferred from reconstructed B-mode image data as it is a proprietary algorithm: we would need the raw channel signal intensity and frequency data to interpret the data. Despite this, relative differences between formulations can be observed as well as the general scattering power, as the transducer used (3 to 7 MHz) falls within the bandwidth of common clinical ultrasound frequencies. The scattering attenuation (represented in units of decibel per centimeter) is approximated using the known physical dimensions displayed in the Terason program and the decibel change in intensity from before and after microbubble injection. It is important to note that a positive value for this quantity represents an increase in intensity from the baseline, which is to be expected from ultrasound contrast agents. We also note that the mean background images are subtracted from each post-injection image to eliminate consistent background artifacts from the transducer.

**Figure 5:**
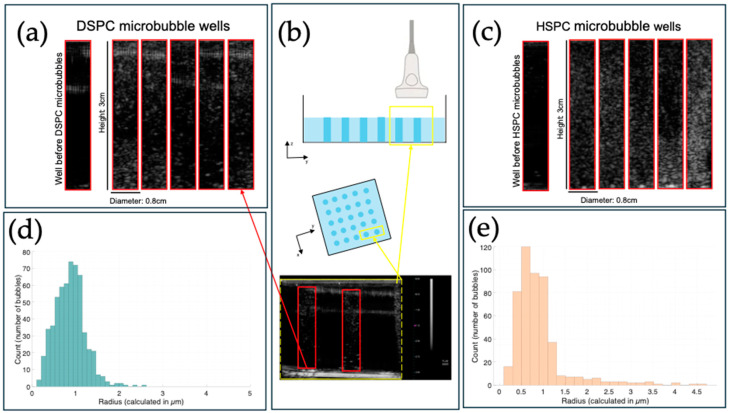
Red boxed area represents B-mode ’wells’ of (**a**) DSPC-lipid and (**c**) HSPC-lipid microbubbles compared next to each other. (**b**) Yellow boxed areas represent total transducer read area, and dotted lines represent unimaged areas. Top image is a side view of the transducer, middle image is a top-side view of transducer, and bottom image is an image saved from the Terason2000 system showing total read area represented. Arrows show where highlighted areas are translated from. The mean background images (shown in Figure 5a,c to the left of the five outlined ‘wells’) are subtracted from each post-injection image to eliminate background artifacts from the transducer. Corresponding histograms for each sample are shown below each acoustic ‘well’ diagram. (**d**) Histogram data representing the population of DSPC-lipid microbubbles shown acoustically in Figure 5a. The concentration of DSPC-lipid microbubbles imaged is 2.67 × 10^8^ ± 5.87 × 10^7^ bubbles/mL (*n* = 5, 24 bins). (**e**) Histogram data representing the population of HSPC-lipid microbubbles imaged acoustically in Figure 5c. The concentration of HSPC-lipid microbubbles imaged is 2.07 × 10^8^ ± 2.55 × 10^7^ bubbles/mL (*n* = 6, 23 bins). Both concentrations are calculated in MATLAB by adapted ‘BubblesizerJ-2.0’.

Figure 5 shows the difference between the DSPC and HSPC microbubble scattering seen in the agarose phantom ‘wells’ on the Terason2000 B-Mode imaging machine. This system is used to test if microbubbles are echogenic and their echogenicity is relative to other microbubbles imaged in the system. Despite being visible at both concentrations (Figure 5), microbubbles do not always produce detectable signals. Each microbubble in a system has its own nonlinear response to an ultrasound wave [16]; by oscillating its shell in response to gas compression, the intensity is enhanced as the incoming ultrasound signal is scattered. The left-most wells shown in Figure 5a,c are representative of a single background well, and not the mean background; the mean of all background wells includes the aberrations introduced by the transducer on the row shown in Figure 5a.

## 4. Discussion

**Figure 6:**
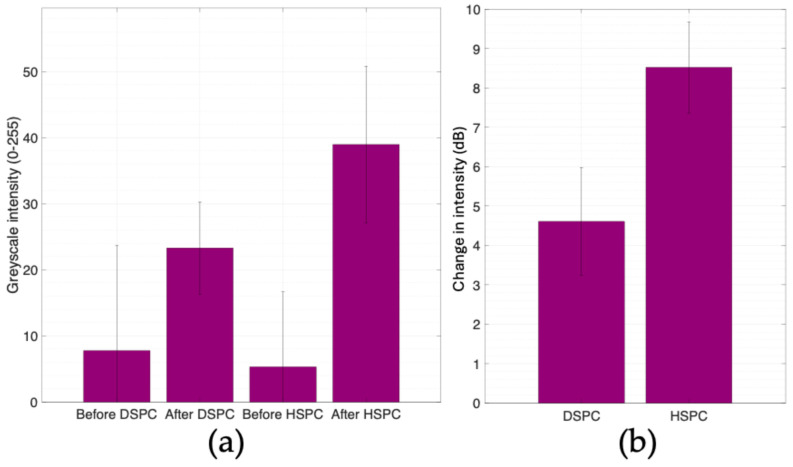
(**a**) Average 8-bit greyscale intensity values of ‘wells’ shown in Figure 5a,c. ‘Before DSPC injection’ and ‘Before HSPC injection’ represent the background measurement with the standard deviation imported from ImageJ. The ‘before injection’ intensity values shown next to the ‘after injection’ values to show the change in intensity from baseline. The standard deviation bars of ‘after’ intensity values in Figure 6a come from the replicate intensity measurements (*n* = 6). (**b**) Estimated change in intensity from intensity changes in observed microbubble ‘wells’ shown in Figure 5. Decibel intensity is calculated by dividing the ‘after’ column greyscale intensity values in Figure 6a by the ‘before’ column in Figure 6a and taking the base-ten logarithm of that ratio; the value is then multiplied by a factor of ten.

Despite the higher mean concentration of microbubbles present in the DSPC sample, the HSPC microbubbles give an increased intensity from the baseline as shown in Figure 6. As previously mentioned, the intensity increase detected through the Terason algorithm is arbitrary and represents the processed signal data. In Figure 6a, the intensity of the wells shown in Figure 5a,c and their standard deviations are plotted. The ‘before’ values represent the mean value of the background intensity prior to injection, which includes the pixel intensity manifested from hardware artifacts. This is why the standard deviation appears to be greater for the background as it is showing the total range of 8-bit values present in the background image. The intensity values of ‘after DSPC’ and ‘after HSPC’ represent the mean intensity after the microbubble injection over five wells. The standard deviation for the groups ‘after DSPC’ and ‘after HSPC’ represents the spread of data from the replicate wells after the background image has been subtracted.

The decibel change in intensity is calculated by taking the following ratio:

$$I_{dB}=10\times \log_{10}\frac{I_{AfterInjection}}{I_{BeforeInjection}}$$

where I_AfterInjection_ and I_BeforeInjection_ represent the greyscale intensities measured by ROIs in ImageJ. A positive change in decibel intensity corresponds to a positive change in intensity post-injection, the desired clinical measurement from the contrast agents.

The approach involves capturing a B-mode image of a microbubble sample along with its size distribution to infer how specific components affect the microbubble structure. This technique is useful during the iterative formulation process for creating complex structures incorporating targeting ligands, polymer brush coatings, crosslinkers, or other functional components that enhance clinical efficacy. By comparing two samples both optically and acoustically, it is possible to assess how different components influence microbubble echogenicity. Characterization methods in the literature commonly employ single- or multi-element ultrasound transducers with tunable frequencies [17,18,19]. We demonstrate that a less specialized clinical device can provide a similar, though more generalized, understanding of microbubble behavior. Greyscale intensity measures have been previously been used to assess changes in muscle fibrosis in Duchenne muscular dystrophy and validated against other clinical imaging techniques such as CT and other ultrasound imaging methods such as quantitative backscatter analysis [20]. This does not imply that the system would not also work with a transducer capable of capturing raw frequency data; rather, the system designed for analyzing microbubbles is effective without requiring access to frequency data.

An additional advantage to having easily obtainable population data is when a specific microbubble size is preferred. Many different methods are reported in the literature to fabricate microbubbles, and often utilize microfluidic devices as a way of controlling the microbubble size [13,21]. The method of gas shaking and lipid film rehydration allows for the rapid formation of monolayer lipid microbubbles at the disadvantage of limited control over population characteristics that could otherwise be finetuned in a microfluidic system. Microfluidic systems often face other issues with lipid formulations such as channel clogging. Size-controlled microfluidic bubbles may provide an advantage in cases where the system being used has a shorter bandwidth since size seems to be critical in the signal output [17,18,22]. As microbubbles become more specialized, it is desirable to have a system where microbubble parameters can be easily monitored during the formulation stage for a clinically modified formula.

For microbubble drug delivery applications, a dual acoustic–optical imaging system can help with determining the threshold for how much pressure to apply to promote inertial cavitation events. A high mechanical index can lead to microbubble cavitation and promote shockwaves and high-velocity jets near the bubble shell as it ruptures, causing the shearing of nearby cells [8]. Unfavorable microbubble events that can lead to cell damage will not happen at lower acoustic pressures, where the shell undergoes typical oscillation events. Thus, when developing a contrast agent that encapsulates a therapeutic agent, it is important to ensure the formulation design does not cause additional harm at the site of microbubble administration.

### Differences Between DSPC and HSPC

DSPC and HSPC microbubbles are similar as both are composed of saturated lipids and contain zwitterionic head groups featuring two oppositely charged functional groups. However, the difference in carbon chain length between the two compounds may account for the variations in bubble size. Longer carbon chains in DSPC may confer increased bubble stability compared to the shorter chains found in HSPC shown in Table 1. This is due to the increased van der Waals forces, which strengthen the intermolecular forces between molecules. Consequently, greater energy is required to disrupt these forces, potentially explaining the larger size of DSPC microbubbles.

**Figure 7:**
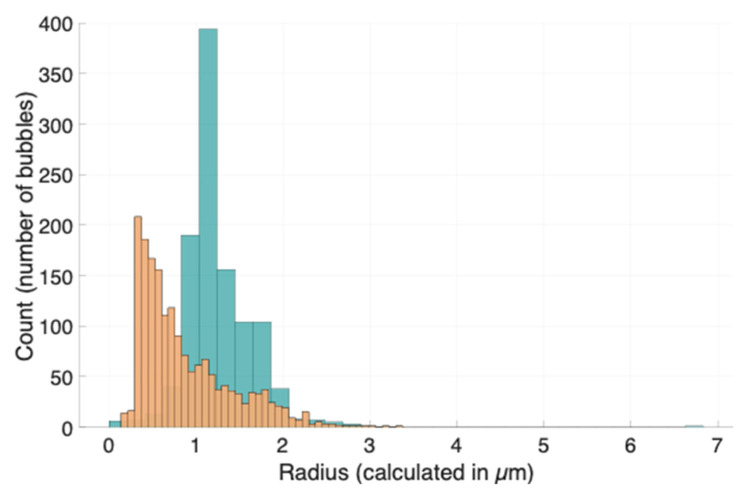
Radii histograms for two independent random samples of microbubbles composed of DSPC lipids (teal) and HSPC lipids (orange), respectively. Two samples from different dates are shown to further explain differences in the distribution between lipid samples. The concentration calculated from ‘BubblesizerJ-2.0’ of the DSPC-lipid microbubbles on the left is 1.71 × 10^8^ ± 4.65 × 10^7^ bubbles/mL; the concentration of the sample of HSPC-lipid microbubbles on the right is calculated as 3.97 × 10^8^ ± 7.95 × 10^7^ bubbles/mL.

A consistent observation of microbubbles produced is that the HSPC lipid often produces a greater range of bubble sizes (compared to the DSPC lipid) at the same molarity. This can be observed in both Figure 5 and Figure 7. In both examples, two independently formulated samples of lipid microbubbles with the same mole fraction (of each respective bulk lipid) showed distributions with a wider range of sizes present for HSPC, the lipid containing the lipid carbon chain length mismatch. This observation is consistent with the fact that, according to Table 1, 11.4% of the total hydrocarbon chains present in our sample of HSPC are 16-carbon instead of 18-carbon. For contrast, DSPC is composed of 18-carbon diacyl phospholipids. This introduced heterogeneity (from HSPC) impacts a percentage of the bubbles formed with lipids of uneven chain lengths. It can be theorized that, by introducing this inhomogeneity, a portion of the population of bubbles is impacted in terms of geometric stability. Introducing lipids with a portion of smaller and inconsistent acyl chains is also theorized to impact the packing structure of the microbubble. The diacyl chain distance (in a phospholipid) presumably influences the degree to which phospholipids can pack [23]. Given a population of lipids shaken with a perfluorocarbon gas, our observations find that, for the uniform PC lipid, there is more homogeneity in the resulting self-assembled microbubble population than in the sample with the differing chain lengths. However, the non-uniform sample produces a ‘range’ of bubbles with an increased surface area of gas–liquid interfaces manifested as an increased amount of smaller-sized bubbles. A polydisperse population has the added benefit of containing multiple resonant frequencies as each subpopulation of bubble sizes has its own resonant frequency [17,18].

As more larger bubbles form (when comparing the HSPC and DSPC microbubbles), there are more frequencies at which the population will experience resonance. The gas–liquid interface of microbubbles allows for the compression of the lipid shell, given that the internal gas experiences expansion and compression while the surrounding liquid does not. Data from centrifugally size-isolated populations of microbubbles suggest that surface viscosity may also play a role in ultrasound-induced shell displacement, and that a lowered surface viscosity of smaller bubbles showed more efficient displacement at higher frequencies [17]. However, it was also mentioned in the same study that samples of different microbubble populations behave as a sum of each individual population and show characteristics of each population at the resonant frequency corresponding to the bubble size [17]. This observation took place when the frequency was controlled for; all acoustic measurements carried out in this study were carried out at the central frequency of the 128-linear element transducer (which falls between 3 and 7 MHz), a closer measurement to what technicians or doctors use in the clinic. Thus, acoustic macroscopic observations carried out to assess clinical viability cannot be used to draw conclusions on individual microbubble behavior. The population histogram from the HSPC sample shown in Figure 5c,e suggests the range of sizes present was wider than that of DSPC and is potentially the main contributing factor of the sample appearing brighter. A study using a tank to collect backscattered signals from Sonovue microbubbles examined whether a predictive model could be developed based solely on population statistics [18]. The authors concluded that the gas volume fraction was a more reliable predictor of the microbubble acoustic output than population parameters alone [18].

Our workflow, which includes a series of observations, provides formulators with tools to prototype and refine different formulations. We demonstrate this through a comparison of two formulations, highlighting the differences observed. Since the bubble size is inversely proportional to the resonant frequency, we show that, using a clinical ultrasound machine, a polydisperse sample containing larger bubbles exhibits a greater change in decibel intensity.

## 5. Conclusions

The functionality of an ultrasound contrast agent comes from its intrinsic physical properties of a gas-filled bubble. To prototype ultrasound contrast agents with any degree of customization, many different formulations need to be tested to ensure they maintain their echogenicity and functionalization. Having a simple yet high-throughput tissue-mimicking phantom for clinically relevant acoustic imaging allows for the benchtop iterative design of acoustically active contrast agents. This advantageous workflow is further supported by the safety and accessibility of portable medical ultrasound machines. It is relatively easy to obtain a clinical ultrasound machine that can be used as a gauge for the relative strength of acoustic contrast agents. Many clinical ultrasound machines come with the ability to obtain raw frequency data that can be used for further analysis to generate frequency response curves. On the drug delivery frontier, having a drug (encased in a microbubble) that can be administered through low-cost, highly accessible, and highly safe ultrasound would enhance clinical availability [8].

## Figures and Tables

**Table 1:** Molecular weight and chain length of lipids used in microbubble formulations. All lipids mentioned have two tails and percentages in subscripts show the percent of population of saturated carbon chains of each length present.

Lipid	1,2-Distearoyl-sn-glycero-3-phosphocholine	mPEG2000-DSPE	L-Alpha-phosphatidyl Choline, Hydrogenated (Soy)
Molecular weight (g/mol)	790.145	2805.497	783.774
Chain length	(18:0)_100%_/(18:0)_100%_	(18:0)_100%_/(18:0)_100%_	(16:0)_11.4%_/(18:0)_88.6%_

## Data Availability

The data generated in this study are presented here in this paper.

## Supplementary Materials

The following supporting information can be downloaded at: https://www.mdpi.com/article/10.3390/bioengineering11121224/s1, Figure S1: BubblesizerJ-2.0 code for brightfield images; Figure S2: BubblesizerJ-2.0 code for phase contrast/darkfield images; Figure S3: MATLAB code for plotting BubblesizerJ-2.0 output.
