# Supplementary material for: Methods for Rapid Characterization of Tunable Microbubble Formulations
Source: Bioengineering (Basel). 2024 Dec 3;11(12):1224. doi: 10.3390/bioengineering11121224 (PMC11673760; doi:10.3390/bioengineering11121224)
Supplement: Supplementary file 1 [file bioengineering-11-01224-s001.zip › bioengineering-3310668-supplementary.pdf]

```
//BubblesizerJ-2.0 Brightfield
//Adapted by Savannah Harpster, 2023

dir = getDirectory("Choose a Directory"); // Ask user to choose a directory
list = getFileList(dir); // Get a list of file names in the chosen directory
for (i = 0; i < list.length; i++) { // Loop through each file in the list
    path = dir + list[i]; // Construct the full path to the current file
    if (endsWith(path,".tif") == true) { // Check if the file is an image
    }
        open(path); // Open the image
                        run("8-bit"); //Convert to 8 bit
                        run("Set Scale...", "distance=(INPUT KNOWN DISTANCE HERE) known=1
unit=micron global"); //Set scale;
                        run("Enhance Contrast","saturated = 0.35 normalize");
                        run("Subtract Background...","rolling = 10 light sliding"); //Subtract background noise
                        run("Gaussian Blur...","radius = 3");
                        run("Maximum...","radius=0.5");
                        run("Invert LUTs");

                        run("Enhance Contrast","saturated = 0.75 normalize");
                        run("Subtract Background...","rolling = 10 sliding");
                        run("Invert LUTs");

                        setOption("BlackBackground", false);
                        setAutoThreshold("MaxEntropy raw");

                        run("Convert to Mask"); //Create mask
                        t = getTitle();

                        run("Options...", "iterations=10000 count=1 edm=8-bit do=[Erode]");
                        run("Options...", "iterations=50 count=1 edm=8-bit do=[Watershed]");//Differentiate
between close particles
                        run("Remove Outliers...", "radius=1 threshold = 1 which=Dark"); //remove tiny artifacts
                        run("Analyze Particles...", "size= (INCLUDE SAME KNOWN DISTANCE HERE -
Infinity pixel circularity= 0.9 - 1 show=Masks display exclude clear include summarize");
                        close(t);
                        q = getTitle();
                        run("Set Measurements...","area shape redirect=None decimal = 5");
                        imagePath = " DESIGNATED OUTPUT FOLDER IN FILE PATH" + i + 1 + ".csv";
                        saveAs("results",imagePath);
                        close(q);
    }
```

Figure S1. BubblesizerJ-2.0 .ijm code for brightfield images of microbubbles. Highlighted text denotes areas where user input is needed for denoting pixel size and output directory.

```
//BubblesizerJ-2.0 Phase/Darkfield
//Adapted by Savannah Harpster, 2023

dir = getDirectory("Choose a Directory"); // Ask user to choose a directory
list = getFileList(dir); // Get a list of file names in the chosen directory
c = getTime();
for (i = 0; i < list.length; i++) { // Loop through each file in the list
   path = dir + list[i]; // Construct the full path to the current file
   if (endsWith(path,".tif") == true) { // Check if the file is an image
   }
        open(path); // Open the image
                        run("8-bit"); //Convert to 8 bit
                        run("Set Scale...", "distance= INSERT KNOWN DISTANCE HERE known=1
unit=micron global"); //Set scale
                        run("Enhance Contrast...","saturated = 0.35 normalize");
                        run("Subtract Background...", "rolling=100 sliding");
                        run("Gaussian Blur...", "sigma=3.75");
                        run("Maximum...","radius=0.5");

                        run("Invert LUTs");
                        run("Subtract Background...", "rolling=100 sliding light");

                        run("Invert LUTs");
                        setOption("BlackBackground", true);
                        setAutoThreshold("MaxEntropy white raw"); //CHANGE AUTOTHRESHOLD FILTER
IF NEEDED HERE
                        run("Convert to Mask"); //Create mask
                        t = getTitle();
                        setOption("BlackBackground", false);

                        run("Options...", "iterations=500 count=8 edm=8-bit do=[Erode]");
                        run("Options...", "iterations=50 count=8 edm=8-bit do=[Watershed]");//Differentiate
between close particles

                        run("Analyze Particles...", "size=INSERT SAME KNOWN DISTANCE HERE-Infinity
pixel circularity = 0.9-1 show=Masks display exclude clear include summarize");
                        close(t);
                        q = getTitle();
                        run("Set Measurements...","area shape redirect=None decimal = 5");
                        r = getTitle();
                        imagePath = "DESIGNATED OUTPUT FOLDER IN FILE PATH" + i + 1 + ".csv";
                        saveAs("results",imagePath);
                        close(r);
                        close(q);
}
```

Figure S2. BubblesizerJ-2.0 .ijm code adapted for darkfield/phase contrast images. Highlighted text denotes areas where user input is needed for denoting pixel size and output directory.

```
%%BubblesizerJ.m
%%Sizes and counts microbubbles
%%Savannah Harpster, 2023

clear
clc
pixelSize = ENTER KNOWN PIXEL RATIO HERE (In units of µm/pixel)

NEXT THREE LINES CALCULATES VOLUME
REPLACE WITH KNOWN PIXEL SIZE AND HEIGHT

totalArea = (pixelSize*2304)^2; %converts pixel area to µm^2
totalVolume = totalArea*10; %units in µm^3; assuming height is 10 microns
totalVolume = totalVolume * (10^-4)^3;%conversion factor to mL (cm^3)

%%Insert directory path to .csv files here (automatically generated by
%%BubblesizerJ)
path = "";
directoryPath = dir(fullfile(path,'*.*'));
tracker = 0;

%Part of code that loops through Results folder
for j = 1:length(directoryPath)
    if ~directoryPath(j).isdir && ~strcmp(directoryPath(j).name, '.DS_Store')
        filePath = fullfile(path, directoryPath(j).name);
        results = readmatrix(filePath);
        results = results(2:end,:);
        x = j-max(tracker);
        Count(x) = 0;
        for i = 1:length(results)
            if results(i,3) >= 0.9 % **WILL NOT WORK IF THERE ARE LESS THAN 5 OBJECTS IN
IMAGE**
                if results(i,6) >= 0.9
                    Count(x,i) = 1; %1 added if value is above the Circularity + Solidity thresholds
                    bubbleRadius{x,i} = num2cell(sqrt(results(i,2)/pi));
                    else
                    Count(x,i) = 0; %0 added if value is below the Circularity + Solidity thresholds
                    bubbleRadius{x,i} = {0};
                end
            end
        end
    else
        tracker(j+1) = tracker(j) + 1;
    end
end

%Part of code that sets empty bubbleRadius cell values ([]) to 0
```

```
    for m = 1:(min(size(Count)))
        for n = 1:max(size(Count))
            if isempty(bubbleRadius{m,n})
                bubbleRadius{m,n} = {0};
            end
         end
    end

 % Part of code that reads through counts and radii, and plots nonzero
 % values
    Count = sum(Count,2);
    concentration = Count/totalVolume;
    meanConcentration = mean(concentration);
    stdConc = std(concentration);
    stdVarConc = 1.96*stdConc/sqrt(length(concentration));
    figure(1);
    bubbleRadius = cell2table(bubbleRadius);
    bubbleRadius = table2array(bubbleRadius);
    bubbleRadius = cell2mat(bubbleRadius);

    bubbleRadiusNONZERO = find(bubbleRadius>0);
    bubbleRadius = bubbleRadius(bubbleRadiusNONZERO); %Indexes at values that are nonzero

    averageRadius = mean(bubbleRadius);
    stdRadius = std(bubbleRadius);
    hold on
    ax = gca;
    ax.FontSize = 36;
    histogram = histogram(bubbleRadius,'NumBins',10);

strConc = ["The mean concentration of bubbles (per mL) is: "+ num2str(meanConcentration,3), "
bubbles/mL with a std. dev. of +/-"+ num2str(stdConc,3) + " bubbles/mL"];
    strRad = ["The average radius of a microbubble from this sample is: " + num2str(averageRadius,3) +
" μm +/- " + num2str(stdRadius,3)];
    dim = [(ax.Position(1)+ax.Position(3))/2,((ax.Position(2)+ax.Position(4))/2)-0.1,0.1,0.1];
    dim2 = [((ax.Position(1)+ax.Position(3))/2),((ax.Position(2)+ax.Position(4))/2)-0.15,0.1,0.1];
    f = annotation('textbox',dim,'String',strConc,'FitBoxToText','on','FontSize',18)
    g = annotation('textbox',dim2,'String',strRad,'FitBoxToText','on','FontSize',16)

set(f,'visible','on','HorizontalAlignment','center','VerticalAlignment','baseline','BackgroundColor','white')

set(g,'visible','on','HorizontalAlignment','center','VerticalAlignment','baseline','BackgroundColor','white')

title('Bubble Radius Histogram','FontSize',24)
ylabel('Count (number of bubbles)')
xlabel('Radius (calculated in μm)')

  hold off
```

```
grid on
grid minor
```

Figure S3. MATLAB code for reading data tabulated from ImageJ (**Figures S1 and S2**). Highlighted areas indicate where user input is required.
